# Out of balance: conflicts of interest persist in food chemicals determined to be generally recognized as safe

**DOI:** 10.1186/s12940-023-01004-8

**Published:** 2023-09-06

**Authors:** Klara Matouskova, Thomas G. Neltner, Maricel V. Maffini

**Affiliations:** 1 Independent Consultant, Bethesda, MD USA; 2 Environmental Defense Fund, Washington, DC USA; 3 Independent Consultant, Frederick, MD USA

**Keywords:** Generally recognized as safe, GRAS, Conflicts of interest, Bias, FDA, Food safety, GRAS expert, US food policy

## Abstract

Manufacturers of chemicals added to food are responsible for determining that the use of their products is safe. There are two major legal definitions of chemicals in food: (1) food additives which includes ingredients and chemicals indirectly entering food from packaging and processing equipment, and (2) generally recognized as safe (GRAS) substances mostly used as ingredients. The law requires food additives to undergo approval by the U.S. Food and Drug Administration (FDA) before they are sold, but it GRAS substances are exempted from pre-market approval. In 1997, FDA created a voluntary program for manufacturers to submit their chemical’s safety determination in the form of a GRAS notice to the agency. Manufacturers make GRAS determinations regardless of whether they voluntarily submit a notice to FDA for review. They rely on their own employees, the employee of a hired consulting firm or a panel of experts, known as GRAS panel, to review the safety information. Because this process determines whether a chemical is safe for use in food, conflicts of interest and biases need to be avoided or minimized to credibly ensure food is safe. Recently, FDA has published guidance for industry on best practices to convene GRAS panels to manage conflicts of interest and reduce biases that have plagued the process. Here, we perform a qualitative assessment of the compliance of GRAS panels with basic elements of FDA’s guidance. We assessed 403 GRAS notices filed by FDA between 2015 and 2020 and identified whether a GRAS panel was convened and by whom, its members, affiliations, and relationships between panelists and panel conveners. Then, we compared FDA’s recommendations against the information included in the notices voluntarily submitted by manufacturers. We found no evidence that GRAS panels have adhered to FDA’s guidance. Panels are populated from a very small pool of professionals; we found that seven panel members alone occupied almost half of all available panel positions and that they often serve together. Against guidance recommendations, *ad-hoc* panels have been substituted by panels with recurring members in hired consulting firms’ payroll. The widespread persistence of conflicts of interest, appearance of conflict and bias in GRAS determinations continue to put the health of Americans at risk and undermine confidence in the safety of food ingredients in the US market. FDA should require notice for all GRAS determinations including how the financial conflicts of interest of those who make these determinations are minimized.

**Supplementary Information:**

The online version contains supplementary material available at 10.1186/s12940-023-01004-8.

## Background

In 1958, the U.S. Congress enacted the Food Additives Amendment (Public Law 85–929, 72 Stat. 1784), requiring food manufacturers to secure FDA’s pre-market approval of food additives. Although common sense indicates that a food additive is something added to food, the term food additive has a specific legal meaning: a substance whose intended use “results or may reasonably be expected to result, directly or indirectly, in its becoming a component of any food or otherwise affecting the characteristics of any food. . ., if such substance is not generally recognized, among experts qualified by scientific training and experience to evaluate its safety, as having been adequately shown. . to be safe under the conditions of its intended use.” (21 U.S.C. § 321(s)). By adding this clause, Congress exempted from the definition of food additives – and FDA’s premarket approval requirements – common ingredients like oils, vinegar, and flour whose uses were considered “generally recognized as safe” (GRAS) [1]. Moreover, FDA has interpreted the law as allowing manufacturers to determine on their own that the use of a substance is GRAS without informing the agency [2].

Whether a food additive or a GRAS substance, it is the manufacturers’ responsibility to determine that the use of their products is safe before they are used in food. Safe or safety is defined to mean that there is “a reasonable certainty in the minds of competent scientists that the substance is not harmful under the intended conditions of use” after considering three factors related to probable exposure, cumulative effect of chemically or toxicologically related substances in the diet, and an adequate margin of safety (21 C.F.R. § 170.3(i)).

Where manufacturers have concluded a substance’s use is GRAS, they can opt to voluntarily submit a GRAS notice to FDA for its review in which case the notice is filed in the agency’s GRAS notice inventory [3]. During the review process, FDA asks clarifying questions usually related to the chemical manufacturing process, exposure assessment or toxicity testing. If FDA is satisfied with the response, it issues a letter stating that it has “no questions” about the GRAS determination. This is an agency opinion, not an approval. If the manufacturer decides not to respond to FDA’s queries, it can request the agency to stop reviewing the assessment and withdraw the notification. Then, FDA issues a “cease to evaluate” letter in response to the request. The manufacturer can market and sale its product without prejudice. Lastly, there are cases where FDA considered that the manufacturer has not demonstrated the substance’s use is GRAS and issues a “no basis” letter. Alternatively, manufacturers can opt to market and sale the ingredient without notifying FDA about the identity of the substance, its safety and uses because it is not required to do so. It has been reported that at least 1000 substances have been determined to be GRAS “in secret” [1].

In its GRAS determination, a manufacturer relies on the opinion of key entities including a panel of experts, commonly known as a GRAS panel, convened either by the manufacturer itself or by a hired third-party (e.g., consulting firm). The other entities consulted are the manufacturer’s own employee or employee of a hired consulting firm [4]. These key entities provide their opinion on whether the available safety data and information about an ingredient – even a novel chemical – are sufficient to conclude that the substance is GRAS under the conditions of its intended use. Convening a GRAS panel is not a requirement however, it is a common practice since the panel may be perceived “as a representative sample of the larger scientific community” and the views of its members as providing “evidence of their respective disciplines’ generally accepted views on a particular question” [5].

In a 2010 report on its review of FDA’s GRAS program, the Government Accountability Office recommended that the agency “develop a strategy” to minimize the potential for conflicts of interest (COI) in “companies’ GRAS determinations”, including issuing guidance for industry on limiting and managing COI as well as requiring information on GRAS panelist independence with the goal of reducing the risk of bias [6]. A 2013 study concluded that financial COI were ubiquitous and that the likelihood that a safety opinion given by regular panelists would be influenced by financial interest was high [4]. A later study also showed that a small group of professionals were frequently hired to serve on GRAS panels [7].

In 2017 FDA published a draft guidance [8] for industry on best practices for convening a GRAS panel as recommended by GAO; the final non-binding guidance has been recently published [5] without substantive changes from the draft. The guidance includes a number of thoughtful and common-sense recommendations that would increase confidence in GRAS determinations, from disclosure of financial conflicts to ensuring that panel members have appropriate and balanced expertise. For example, FDA recommends avoiding including panel members who have their own work used as evidence for safety of the substance under review, persons that consistently and strongly advocate for specific views or positions on scientific issues relevant to safety assessment, and prior employees of the manufacturer of the substance under review. Furthermore, the agency recommends that ad hoc rather than standing panels are convened to avoid for the development of a group perspective over time. Lastly, the guidance also includes clear recommendations about more traditional - financial - conflicts of interest [5].

The goal of this Commentary was to identify whether GRAS notices filed by FDA after the publication of its draft guidance on best practices for convening a GRAS panel have adhered to the agency’s recommendations. We identified the key entities manufacturers relied on in their GRAS determinations, how often members serve in panels, frequency of members participation in the same panel, and panel members’ relationship with third-party GRAS panel conveners. We analyzed this information and compared it against FDA recommendations.

## Main text

### Methods

We assessed 403 GRAS notices FDA filed and completed between calendar years 2015 and 2020 from the agency’s GRAS Notices Inventory [3]. This timeframe roughly followed the last review of GRAS notices by Hanlon et al. [7] who reviewed the first 600 notices.

From each GRAS notice we extracted: (1) the notice number, a unique identifier given by FDA; (2) the substance’s identity; (3) name of the notifier, typically the manufacturer of the substance; (4) if there was a GRAS panel, the number of members and their names and affiliations, and if it was convened by a third-party.

For each unique panel member, we examined: (1) frequency of participation in GRAS panels, (2) their relationship with the panel convener (e.g., notifier or third-party), and (3) how often members served together on the same panel. We defined frequent relationship as serving together five or more times; this is an indication of the size of the pool of experts, strength of relationship between panelists and risk of bias.

We then performed a qualitative assessment of the compliance of GRAS panels with the basic elements of the FDA’s best practices guidance. We compared notices submitted in 2015–2017 (before the guidance was published) to those submitted in 2018–2020 (after the guidance) and assessed the composition of the panels, specifically focusing on COI, appearance of conflict, and risk of bias.

### Findings

#### Most companies voluntarily submitting GRAS notices to FDA for review relied on panels of experts to provide an opinion on their GRAS safety determination

On average a notifier relied on a GRAS panel in 57% of the 403 GRAS notices reviewed by FDA between 2015 and 2020 (Table 1). See supplemental information for details and links to the notices.

**Table 1:** Key entities a notifier relies on when determining a substance’s use is GRAS.

	2015 (n = 51)^1^	2016 (n = 65)	2017 (n = 70)	2018 (n = 73)	2019 (n = 67)	2020 (n = 77)	Average (total = 403)
GRAS panel organized by notifier or third-party	60%	70%	61%	45%	49%	62%	**57%**
Employee of notifier	26%	19%	33%	32%	38%	32%	**30%**
Employee of third-party	14%	11%	6%	23%	14%	6%	**13%**

^1^ Number of GRAS notices filed by FDA in the given year

Our analysis of the GRAS panel convened during the three years after the draft guidance was published (2018–2020) shows that none of them follow FDA’s recommendations. We were unable to discern any difference between panels convened before and after the publication of the best practice guidance in 2017 that would indicate adherence to FDA’s recommended best practices.

#### Assessment of the GRAS panels showed that individuals from a small pool of experts were frequent panel members with conflicts of interest, appearance of conflicts, and a high risk of bias contrary to FDA’s guidance to minimize those issues

There was a total of 232 GRAS panels convened, an average of 38.5 panels per year. The average number of members on a panel was 3.2 (range 2–7). The total number of panel positions in the 2015–2020 period was 732 with an average of 122 panel positions per year.

**Fig. 1:**
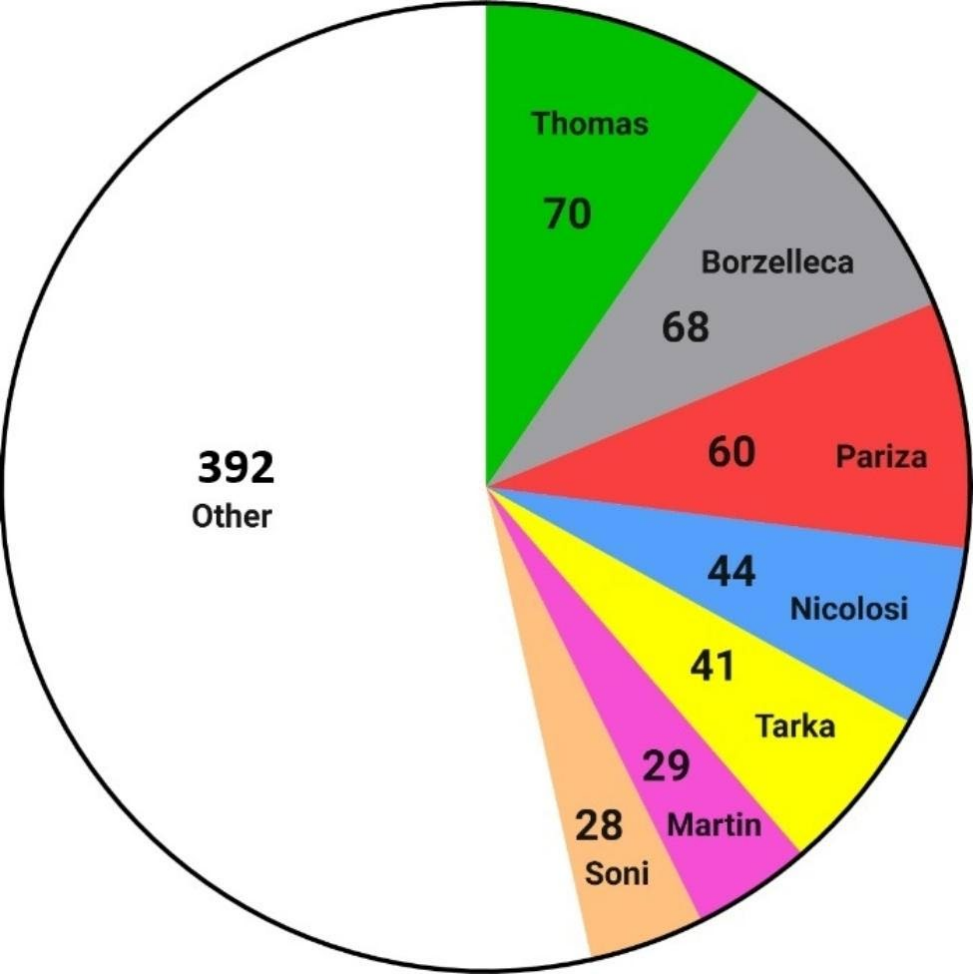
Members serving on GRAS panels were selected from a small pool of experts. Seven individuals were frequently hired to serve in the 2015–2020 period. Their combined participation amounted to 46% of all available panel positions ( 340 out of 732 ). The remaining 54% were populated from a pool of over a hundred panelists. Numbers indicate panel participation

We found that seven individuals dominated the panels and they often served together. Drs. J.A. Thomas, J.F. Borzelleca, M.W. Pariza, R. Nicolosi, S. Tarka, R.L. Martin and M.G. Soni were frequently selected to participate in GRAS expert panels and combined, they accounted for 46% (339) of the 732 panel positions available (Fig. 1).

In addition, these seven panelists often served together as shown on Table 2. Thomas, Borzelleca, Pariza and Nicolosi served together with high frequency. For instance, Borzelleca served with Pariza 28 times, Nicolosi 25 times and Thomas 20 times.

The frequent participation of a cadre of people has been reported before [4] and some authors [7] have called them “most prolific GRAS expert panelists”, a description at least one expert has fully embraced in marketing his business [9]. However, these widespread issues continue to undermine the credibility of GRAS panels opinions and emphasize the need for FDA’s best practice recommendations to be strictly followed. The fact that the same seven individuals have been members of GRAS panels for more than two decades is a strong indication that they often agree with the notifier’s GRAS determination thus increasing their chances to be hired again. Therefore, GRAS panel participation likely becomes a source of income and leads to strong financial conflicts of interests. Limiting and managing financial conflicts of interest should be the most straightforward of FDA’s recommendations to meet since it is readily identified.

**Table 2:** Number of GRAS panels convened in 2015–2020 in which the seven most frequently hired panelists served together

.	Thomas	Borzelleca	Pariza	Nicolosi	Tarka	Martin	Soni
Thomas	.	20	15	25	13	13	12
Borzelleca	20	.	28	25	2	0	2
Pariza	15	28	.	10	10	1	3
Nicolosi	25	25	10	.	2	0	0
Tarka	13	2	10	2	.	1	3
Martin	13	0	1	0	1	.	20
Soni	12	2	3	0	3	20	.

FDA guidance also has recommendations to ensure the credibility of the GRAS panel report including appearance of conflicts of interest and bias, and the agency recommends that conveners of a panel “establish and implement a written GRAS panel policy” addressing these and other procedural issues like documenting selection and vetting of members, and the deliberations. From our analysis, we were unable to find evidence that the conveners have applied such a policy. Although bias is hard to identify, it is difficult to conceive that individuals that have been panel members for decades and often serve together have not developed certain cognitive patterns which will create bias [10, 11]. If being a GRAS panel member is a major source of income, the potential for financial gain can also increase the risk of bias at the individual and group level [12, 13].

#### FDA guidance recommends that GRAS panels are convened on an ad-hoc basis to ensure representation of expertise and limit bias. In reality, ad-hoc panels are being replaced with standing panels populated with individuals financially tied to consulting firms

**Fig. 2:**
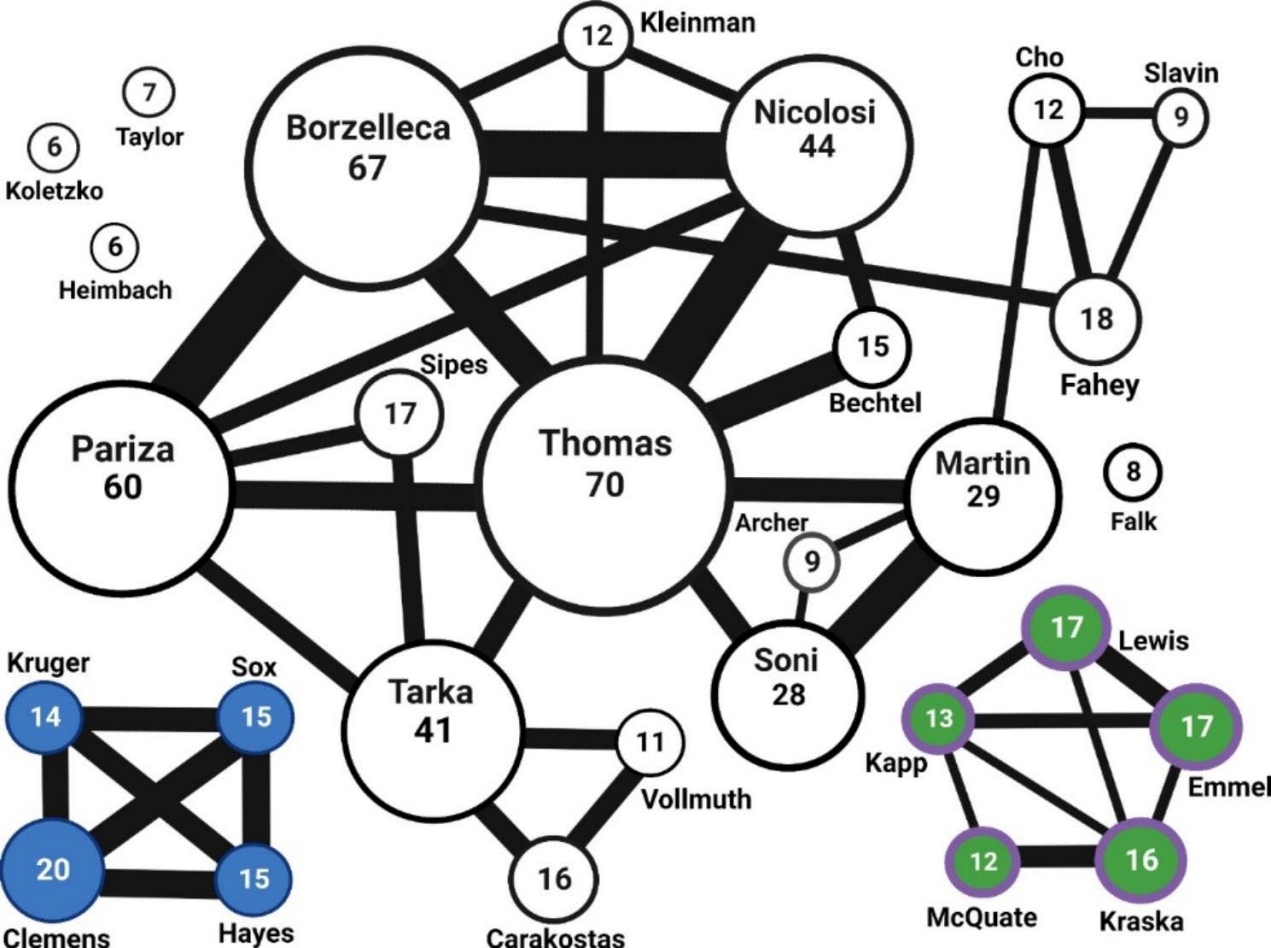
Schematic representation of the 29 individuals that served on six or more GRAS panels between 2015–2020. The size of the circle illustrates the frequency of participation; the number inside each circle indicates the exact participation. A line between two circles indicates that the individuals served on the same panel at least five times; the thicker the bar the higher the frequency of serving together. The two self-contained clusters represent individuals that appear together in panels convened by a specific consulting firm: Spherix Consulting Group, Inc. (blue circles) and GRAS Associates, LLC (green circles)

FDA, citing a National Academies of Science Medicine and Engineering report [14], states that “longer and closer associations increase the scope and therefore the risk” for undue influence. Figure 2 shows that 29 individuals served on six or more GRAS expert panels between 2015 and 2020. Most panelists appeared to be hired by multiple third-parties, however, we identified two distinct clusters of individuals closely related to a single party. Consulting firms Spherix Consulting Group, Inc. and GRAS Associates, LLC, appear to consistently populate GRAS panels with the same individuals. For instance, Drs. Kruger, Sox, Hayes, and Clemens appeared to only be associated with Spherix. Similarly, Kapp, McQuate, Kraska, Lewis and Emmel have a near exclusive relationship with GRAS Associates. While some of these recurring experts are listed as senior external consultants, they rarely participate in panels convened by other parties.

FDA’s guidance recommends convening of ad hoc rather than standing GRAS panels to optimize the applicable expertise and reduce bias. However, we identified closely related clusters where the third-party hired to manage the GRAS notice (i.e., organizing safety information, hiring experts, communicate with FDA), consistently convened the same panel members. This practice is contrary to FDA’s recommendation to avoid standing panels. Equally concerning is the fact that the panels’ organizers are also participants. FDA strongly discourages that those organizing panels participate as a member and recommends that the organizers “not be members of a GRAS panel, because such individuals generally would have a conflict of interest due to a direct and predictable financial interest in the outcome of the panel’s deliberations” [5]. Here is a clear example demonstrating the lack of compliance: panels organized by the consulting firm Spherix Consulting Group consistently relied on Drs. Clemens, Hayes, Sox and Krueger. We noted that Krueger is a managing partner at the firm [15] and she is also a frequent member in GRAS panels convened by her firm. The other experts are listed as senior external consultants although they rarely participate in GRAS panels convened by other firms. Soni, from Soni and Associates, Inc. [9] is another example of GRAS panel organizer participating as a member.

#### FDA’s well-intentioned guidance to convene GRAS panels does not include measures to limit the more concerning conflicts of interest that occur when companies rely on their employees or hired consultants

As shown in Table 1, up to 39% of the safety decisions did not involve a GRAS panel or a third-party which means that notifiers (i.e., substance manufacturers) relied on an opinion provided by their own employee to make a GRAS determination. In this case, the likelihood of the decision to be unduly influenced by the financial interest of a notifier is highest [14] and an employee of the notifier is also the least independent reviewer of a safety assessment, completely undermining its credibility. Lastly, in up to 23% of the notices, a notifier relied on employees of a third-party hired to conduct the GRAS safety evaluations. These people typically have significant bias and conflicts of interest because positive decisions help their employer and its client secure more business.

## Conclusions

In this Commentary, we show that bias and conflicts of interest keep shaping food safety in the United States. Safety decisions continue to be made by individuals with strong financial conflicts of interest such as ingredient manufacturers and supported by experts that have made GRAS panel participation their source of income for more than 20 years. Even worse, the safety determination is supported by employees of the company profiting from sales of the ingredient, or employees of a hired third-party with a strong interest in looking out for its client. It is of great concern that a cadre of people is deciding on the safe use of substances that could potentially impact the health of millions of Americans [16]. And it is hard to fathom that there is such a small pool of experts qualified and available to provide independent opinions on food substances safety assessments that reflect the broader scientific community.

FDA’s guidance to convene GRAS panels is a well-intentioned document with excellent recommendations aimed at bringing credibility to GRAS safety determinations by ensuring the biases, financial conflicts of interest and appearance issues have been managed and panel members vetted for independence and expertise. However, FDA repeatedly says that a “GRAS panel is just one mechanism” that can be used to support a GRAS conclusion, implicitly acknowledging that a GRAS conclusion reached by an employee of the manufacturer is similarly trustworthy. A GRAS conclusion supported by the opinion of an employee is perhaps the worst case of undermining confidence in the safety of food ingredients. And these concerns are further aggravated when the manufacturer decides to bypass FDA review of the GRAS determination. FDA should make clear that the best practices apply to everyone involved in the safety evaluation process, and not just GRAS panels.

Lastly, there has been little improvement to the GRAS system since the GAO recommended major changes to rebuild consumer confidence in the safety of the food supply 13 years ago. The guidance is a good step forward in meeting that goal. But even more important is for FDA to require that it be notified of all GRAS determinations.

### Electronic supplementary material

Below is the link to the electronic supplementary material.

Supplementary Material 1

## Data Availability

All data generated or analyzed during this study are included in this published article [and its supplementary information files].

## List of Abbreviations

GRAS: Generally Recognized as Safe
FDA: Food and Drug Administration
U.S.C.: United States Code
C.F.R.: Code of Federal Regulation
COI: Conflicts of Interest
GAO: Government Accountability Office
GRN: GRAS notice
LLC: Limited Liability Company
